# Cardiothoracic Imaging Findings in Obesity Hypoventilation Syndrome (Pickwickian Syndrome): A Case Series

**DOI:** 10.7759/cureus.114686

**Published:** 2026-08-17

**Authors:** Munkhsuvd Chimed, Anujin Baljinnyam, Shreya Mahasenan, Ismail M Kabakus, Jeremy R Burt

**Affiliations:** 1 Radiology, Intermed International University of Health and Welfare (IUHW) Hospital, Ulaanbaatar, MNG; 2 Radiology, The University of Utah, Salt Lake City, USA; 3 Radiology, Medical University of South Carolina, Charleston, USA

**Keywords:** cardiothoracic imaging, mosaic attenuation, obesity hypoventilation syndrome, pickwickian syndrome, pulmonary hypertension

## Abstract

This case series describes a recognizable multimodality cardiothoracic imaging pattern in five patients with obesity hypoventilation syndrome (OHS), also known as Pickwickian syndrome. All five patients had obesity, obstructive sleep apnea (OSA), daytime hypoxemia, and hypercapnia and underwent cardiothoracic imaging. The mean age was 56.8 ± 19 years, the mean BMI was 46.5 ± 7.4 kg/m², and 60% were male. Chest radiography (CXR) demonstrated enlargement of the interlobar pulmonary artery to greater than 16 mm and central pulmonary arterial dilatation. CT showed central pulmonary artery enlargement with relative attenuation of the peripheral pulmonary arterial branches. Mosaic lung attenuation was present in most cases. Cardiomegaly was present on 75% of the available chest radiographs, and right ventricular enlargement was present on CT in 60% of patients. When performed, echocardiography was limited or nondiagnostic. One patient underwent right heart catheterization, which confirmed mild pulmonary hypertension. Cardiac magnetic resonance (CMR) imaging, which was performed in only one patient, demonstrated main pulmonary artery enlargement and mildly reduced right ventricular systolic function without an alternative diagnostic explanation. In patients with obesity, sleep-disordered breathing, daytime hypercapnia, and hypoxemia, a multimodality imaging pattern comprising central pulmonary artery enlargement, relative peripheral vascular attenuation, mosaic lung attenuation, and variable right-sided cardiac remodeling should raise suspicion for possible OHS-associated pulmonary vascular disease and prompt further cardiopulmonary evaluation.

## Introduction

Obesity hypoventilation syndrome (OHS), also known as Pickwickian syndrome, is characterized by the combination of obesity, sleep-disordered breathing, and daytime hypercapnia after the exclusion of other causes of alveolar hypoventilation [1-4]. Diagnostic criteria include a BMI ≥30 kg/m², daytime arterial carbon dioxide tension (PaCO₂) ≥45 mmHg, and coexisting sleep-disordered breathing.

The pathophysiology of OHS is multifactorial and encompasses obesity-related mechanical respiratory impairment, altered central ventilatory drive, and abnormalities associated with sleep-disordered breathing [2-4]. Excessive fat deposition around the abdomen and chest wall reduces lung volumes, limits diaphragmatic excursion, and impairs respiratory mechanics, leading to inadequate ventilatory compensation and resulting in chronic carbon dioxide retention and hypoxemia. Persistent hypoventilation and recurrent nocturnal oxygen desaturation contribute to pulmonary vasoconstriction, vascular remodeling, and secondary right heart strain [2,5,6]. Approximately 90% of patients with OHS have coexisting obstructive sleep apnea (OSA), often in a severe form, making upper airway obstruction a key factor in both nocturnal and daytime gas-exchange abnormalities [1,2].

Over the past decade, the prevalence of severe obesity has increased worldwide. Previous literature on pulmonary hypertension associated with OHS has described imaging findings such as central pulmonary artery enlargement, right heart remodeling, and mosaic lung attenuation as indicators of possible pulmonary vascular disease [5-8]. However, a comprehensive description of the characteristic multimodality cardiothoracic imaging pattern in OHS, particularly in patients without pleuroparenchymal lung disease, is lacking.

We present a five-patient case series illustrating a recurring cardiothoracic imaging pattern in OHS, characterized by central pulmonary artery enlargement, relative attenuation of the peripheral pulmonary arterial branches, mosaic lung attenuation in the absence of pleuroparenchymal lung disease, and right-sided cardiac remodeling.

Early recognition of this imaging pattern may help clinicians identify possible OHS-associated pulmonary vascular involvement and prompt timely cardiopulmonary evaluation.

## Case presentation

Methods

This case series includes five patients evaluated at the University of Utah between January 1, 2010, and January 1, 2025, who met the diagnostic criteria for obesity hypoventilation syndrome: BMI ≥30 kg/m², daytime arterial carbon dioxide tension (PaCO₂) ≥45 mmHg, sleep-disordered breathing confirmed by sleep testing, and exclusion of other causes of alveolar hypoventilation. Patients were identified through a retrospective search of the radiology database. All imaging studies were reviewed by a single radiologist.

Summary statistics

Summary statistics were generated using standard descriptive methods. Continuous variables were summarized using the mean, standard deviation, median, IQR, and range. Binary variables were summarized using counts and percentages, with the denominator representing the number of non-missing observations. Missing data were quantified for each variable.

Case 1

A 35-year-old man with class III obesity and a BMI of 55.5 kg/m² presented with excessive daytime sleepiness and severe hypoxemia, with an oxygen saturation of 80% on room air. He had no previous history of lung disease. Sleep testing confirmed OSA. ABG analysis demonstrated daytime hypoxemia, defined as a partial pressure of arterial oxygen (PaO₂) <70 mmHg, and hypercapnia, defined as a partial pressure of arterial carbon dioxide (PaCO₂) ≥45 mmHg. Polycythemia was present. Pulmonary function tests (PFTs) were not performed.

**Figure 1:**
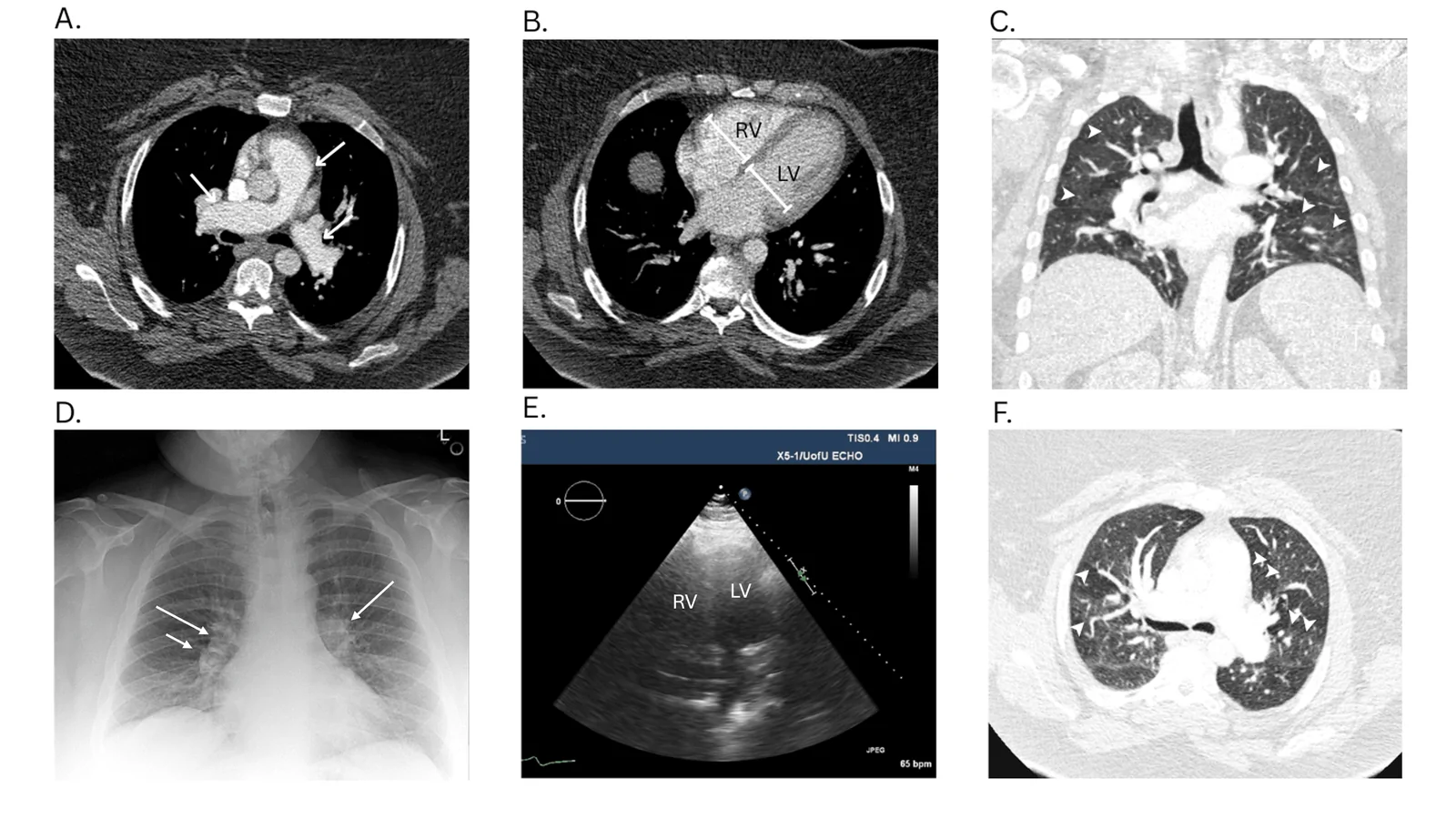
Imaging findings in Case 1. Axial CT images show enlargement of the central pulmonary arteries (A, arrows) and right ventricular (RV) enlargement (B). Coronal and axial CT images displayed in lung windows (C and F) demonstrate moderate, diffuse, patchy mosaic attenuation (arrowheads) and relatively small peripheral pulmonary arteries. A frontal chest radiograph (D) shows enlarged branch pulmonary and interlobar arteries with attenuated peripheral pulmonary vasculature. An apical four-chamber transthoracic echocardiographic image (E) provides limited visualization of the right heart and left atrium.

Chest radiography (CXR) demonstrated enlargement of the central pulmonary arteries, relative attenuation of the peripheral pulmonary arterial branches, and cardiomegaly, without evidence of underlying lung disease. The right interlobar pulmonary artery measured 22 mm in diameter. Contrast-enhanced CT of the chest demonstrated a main pulmonary artery trunk diameter of 33 mm (reference upper limit of normal, 29 mm), markedly enlarged central pulmonary arteries with comparatively small peripheral branches, right ventricular (RV) enlargement relative to the left ventricle (LV), and a moderate mosaic attenuation pattern in the pulmonary parenchyma. There was no evidence of pleuroparenchymal lung disease or pulmonary arterial filling defects. The constellation of findings was suspicious for pulmonary hypertension in the setting of obesity hypoventilation syndrome (Figure 1). Echocardiographic assessment was inconclusive because of suboptimal visualization of the right heart and pulmonary arteries. Cardiac magnetic resonance (CMR) imaging was not performed.

Case 2

A 62-year-old man with class II obesity and a BMI of 36.7 kg/m² presented with shortness of breath. He had no prior history of lung disease. Sleep testing confirmed OSA. ABG analysis demonstrated daytime hypoxemia and hypercapnia. PFT results were normal, and polycythemia was not present.

**Figure 2:**
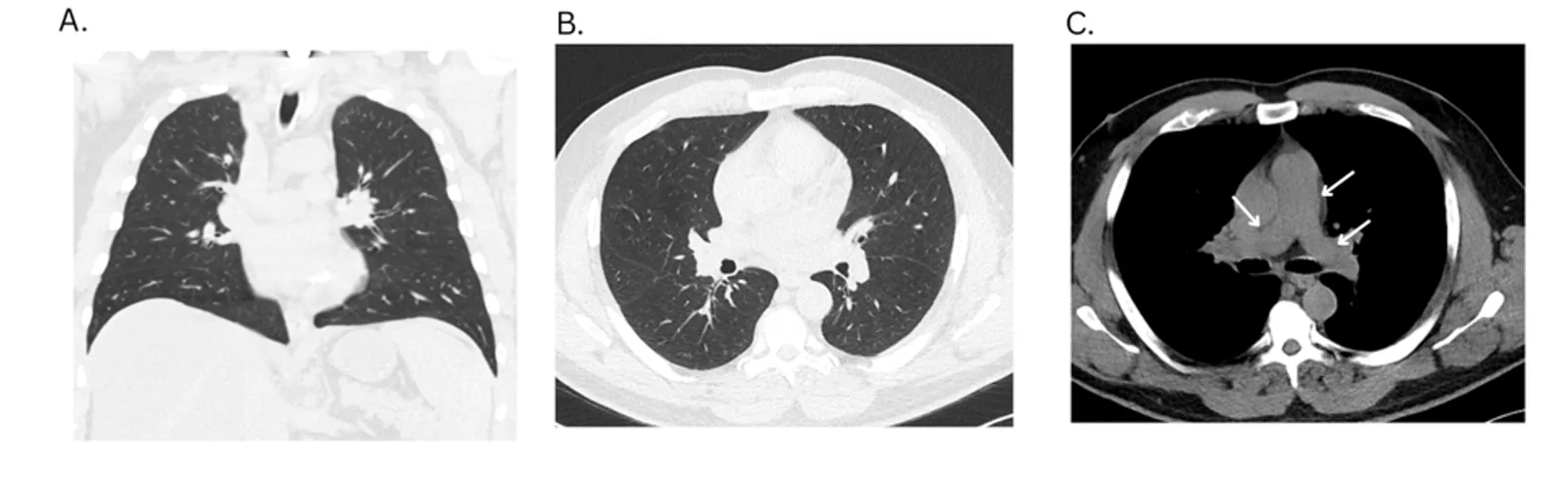
CT imaging findings in Case 2. Coronal and axial computed tomography (CT) images show enlarged central pulmonary arteries (C, arrows) and relative attenuation of the peripheral pulmonary arteries (A and B). Note the absence of mosaic lung attenuation.

CXR demonstrated enlargement of the central pulmonary arteries, with relative attenuation of the peripheral pulmonary arterial branches. The right interlobar pulmonary artery measured 19 mm in diameter. No cardiomegaly or pleuroparenchymal lung disease was identified. Unenhanced CT of the chest demonstrated enlarged central pulmonary arteries, relative attenuation of the peripheral pulmonary arterial branches, normal cardiac chamber sizes, no mosaic lung attenuation, and no pleuroparenchymal lung disease (Figure 2). Echocardiography and CMR imaging were not performed.

Case 3

A 74-year-old man with class III obesity and a BMI of 44.8 kg/m² presented with persistent shortness of breath. He had no history of lung disease. Sleep testing confirmed OSA. Arterial blood gas (ABG) analysis demonstrated daytime hypoxemia and hypercapnia. Polycythemia was not present. PFTs demonstrated a moderate restrictive ventilatory defect without airway obstruction.

**Figure 3:**
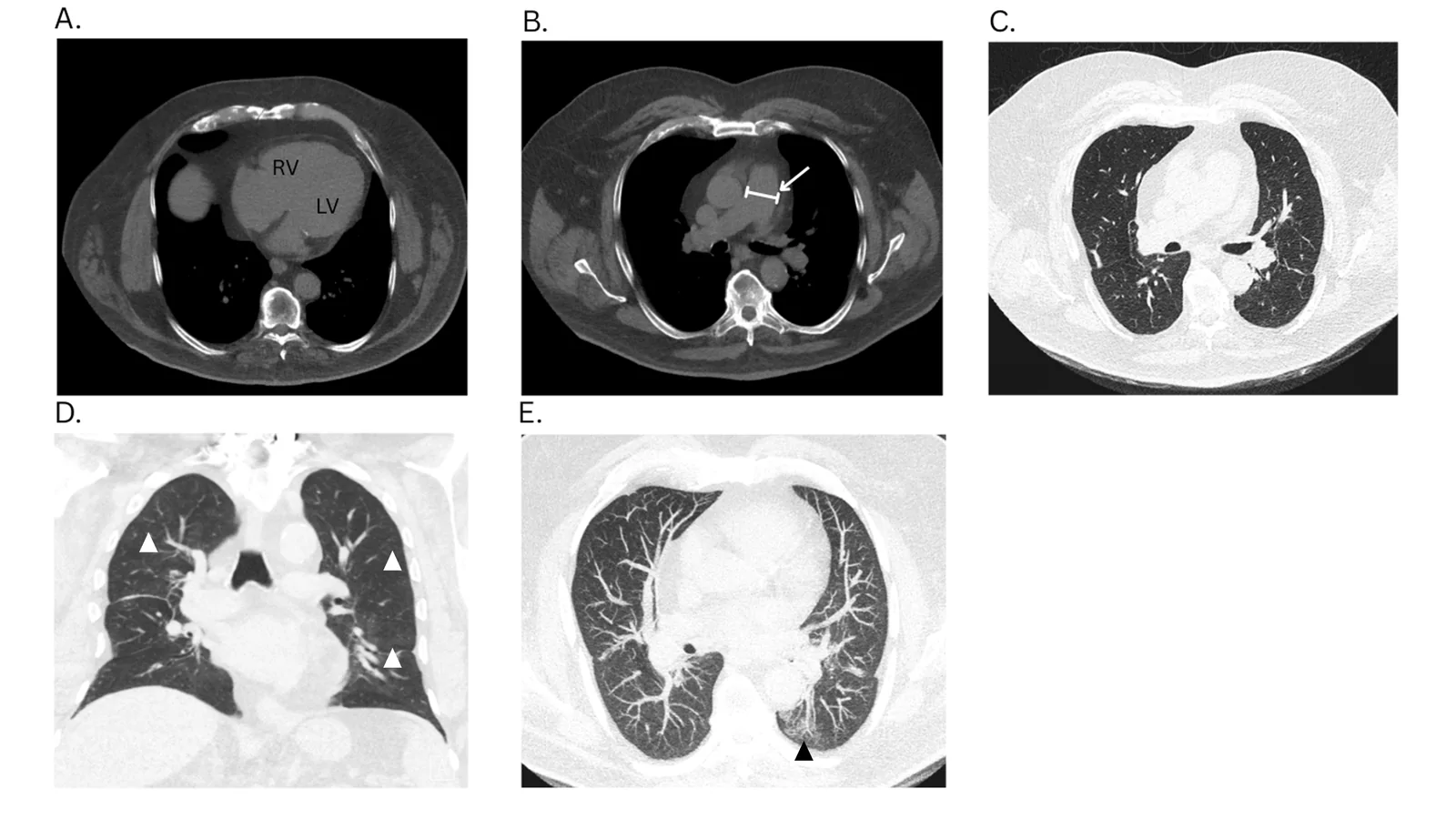
CT imaging findings in Case 3. Axial and coronal computed tomography (CT) images show right ventricular (RV) and central pulmonary artery enlargement (A and B, arrow), abrupt tapering of the small peripheral pulmonary arteries (C-E), and diffuse, patchy mosaic attenuation (D and E, arrowheads). Panel E is a maximum-intensity projection image.

CXR demonstrated enlargement of the central pulmonary arteries, with relative attenuation of the peripheral pulmonary arterial branches. The right interlobar pulmonary artery measured 20 mm in diameter. Unenhanced CT of the chest demonstrated enlargement of the central pulmonary arteries with comparatively smaller peripheral branches, RV enlargement, and a mild mosaic attenuation pattern in the pulmonary parenchyma, without evidence of pleuroparenchymal lung disease (Figure 3). Echocardiography and CMR imaging were not performed.

Case 4

A 39-year-old woman with class III obesity and a BMI of 43.7 kg/m² presented with chronic shortness of breath. She had a history of congenital aortic surgery but no known lung disease. Sleep testing confirmed OSA. ABG analysis demonstrated daytime hypoxemia and hypercapnia. Polycythemia was not present. PFTs demonstrated a moderate restrictive ventilatory defect without airway obstruction.

**Figure 4:**
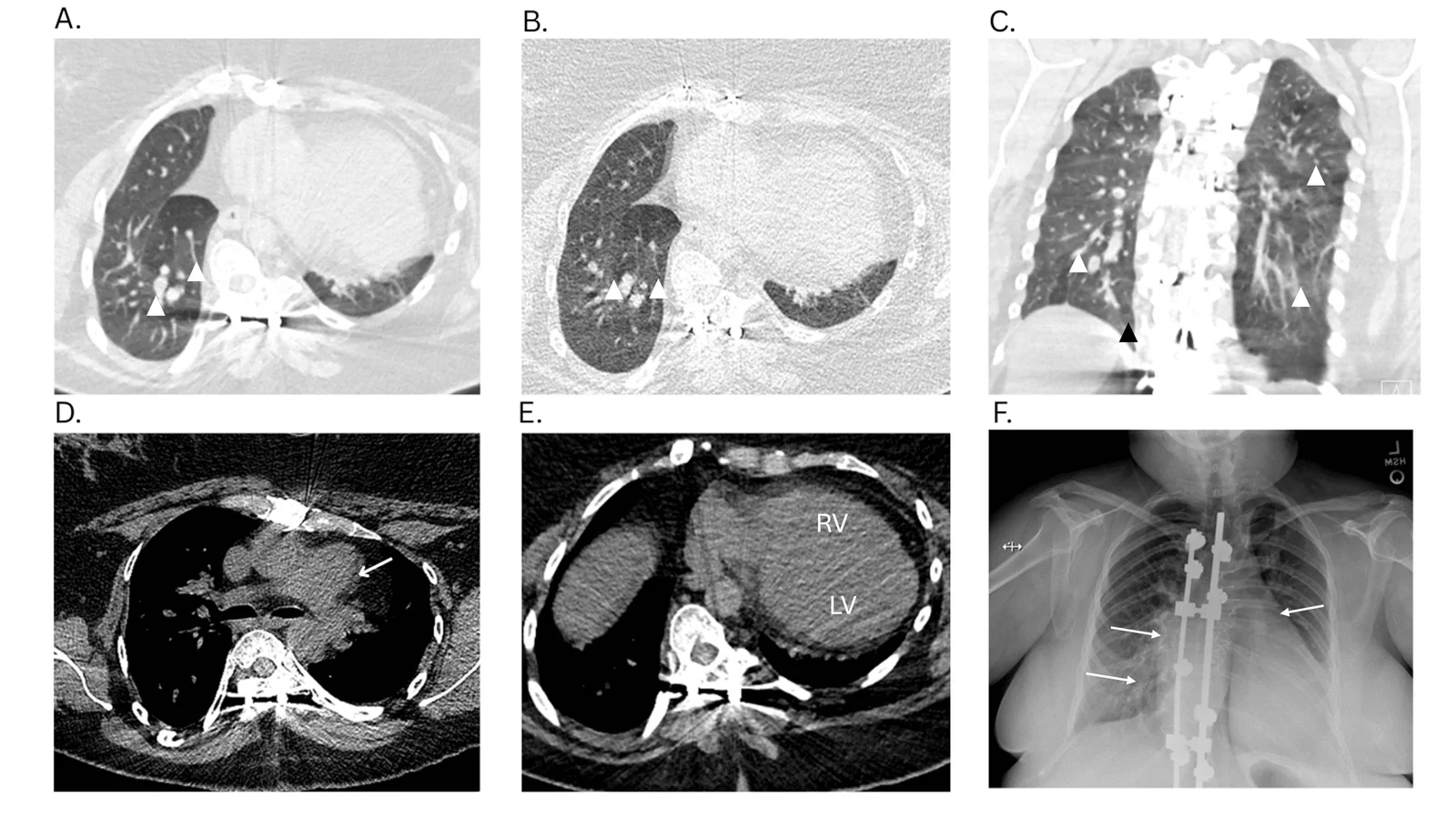
Imaging findings in Case 4. Axial and coronal computed tomography (CT) images show mild mosaic attenuation (arrowheads) with relative attenuation of the peripheral pulmonary arteries (A-C), enlargement of the main pulmonary artery (D, arrow), and biventricular enlargement (E). The CT images are limited by artifacts from spinal fusion hardware and sternotomy wires. A pectus excavatum deformity is also present. A frontal chest radiograph (F) demonstrates a large body habitus, cardiomegaly, and enlargement of the hilar and interlobar pulmonary arteries.

CXR demonstrated enlargement of the central pulmonary arteries and cardiomegaly, without evidence of pleuroparenchymal lung disease. The right interlobar pulmonary artery measured 17 mm in diameter. Unenhanced CT of the chest demonstrated enlarged central pulmonary arteries with small peripheral branches, mildly enlarged ventricles, and a mild mosaic attenuation pattern, without pleuroparenchymal lung disease (Figure 4).

**Figure 5:**
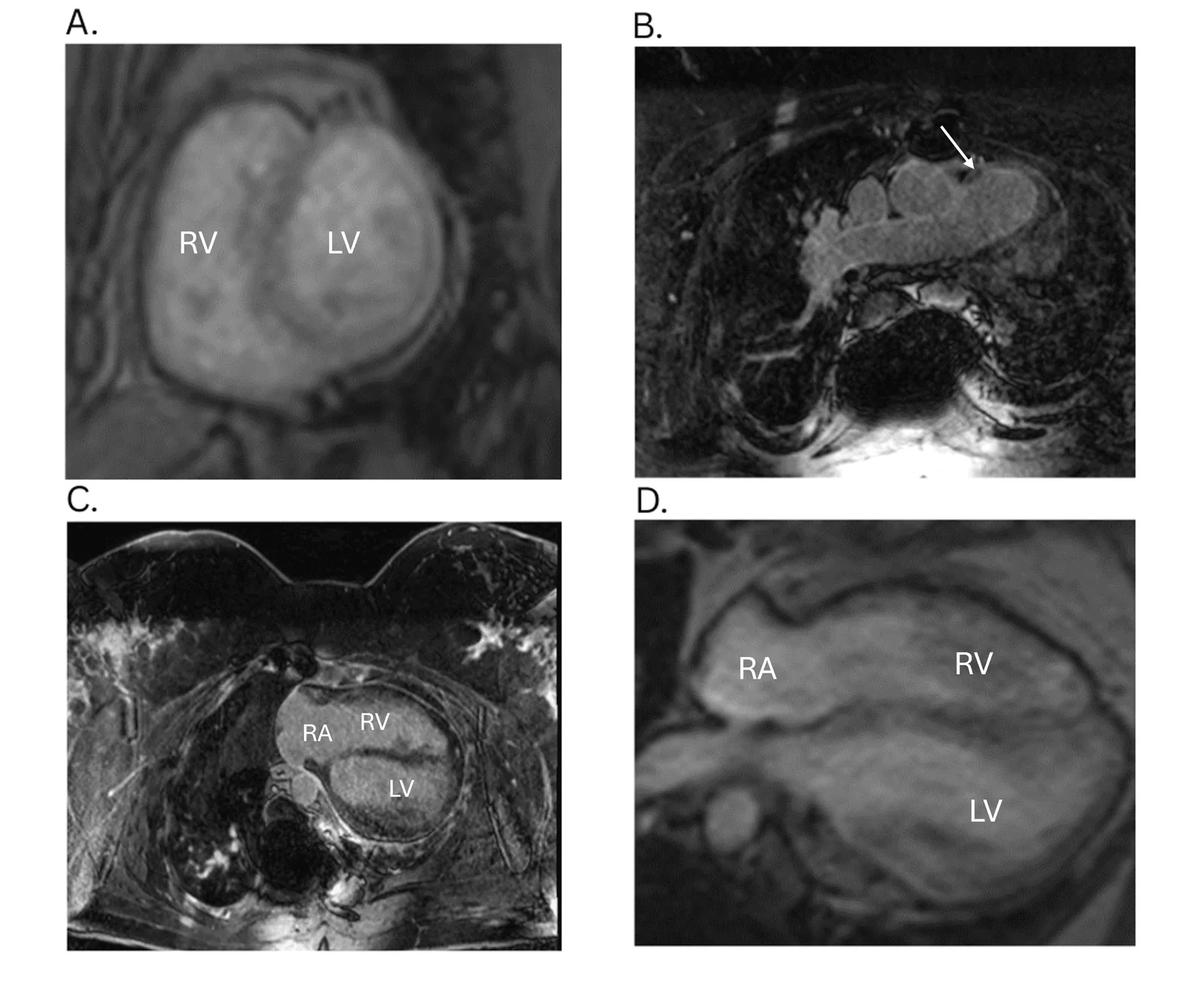
Cardiac MRI findings in Case 4. Cardiac magnetic resonance (CMR) images include a real-time spoiled gradient-echo short-axis image (A), 3D delayed postcontrast axial images (B and C), and a balanced steady-state free-precession four-chamber long-axis image (D). The images demonstrate main pulmonary artery and biventricular enlargement, mildly decreased right ventricular systolic function (ejection fraction = 40%), and normal left ventricular systolic function (ejection fraction = 55%). No delayed myocardial enhancement is present. RA: Right atrium; RV: Right ventricle; LV: Left ventricle; Arrow points to the main pulmonary artery.

Echocardiographic assessment of the right heart was nondiagnostic because of poor sonographic windows. CMR imaging demonstrated an enlarged main pulmonary artery, mildly enlarged ventricles, mildly decreased RV systolic function, normal left ventricular systolic function, normal right and left ventricular wall thickness, and no delayed myocardial enhancement or valvular disease (Figure 5). Right heart catheterization confirmed mild pulmonary arterial hypertension with a vasodilator response to adenosine. Given the patient’s history of congenital aortic surgery, congenital cardiovascular anomalies involving the pulmonary arteries, including an anomalous pulmonary arterial origin or cardiovascular shunt, could not be excluded without contrast-enhanced imaging or additional cardiac evaluation.

Case 5

A 75-year-old woman with class III obesity and a BMI of 51.9 kg/m² presented with chronic shortness of breath. She had no history of lung disease. Sleep testing confirmed OSA. ABG analysis demonstrated daytime hypoxemia and hypercapnia. Polycythemia was not present. PFTs were not performed.

CXR demonstrated enlargement of the central pulmonary arteries, pruning of the peripheral pulmonary vasculature, and cardiomegaly, without evidence of pleuroparenchymal lung disease. The right interlobar pulmonary artery measured greater than 21 mm in diameter.

Unenhanced CT of the chest demonstrated a main pulmonary artery diameter of 47 mm, with small peripheral branches, RV enlargement with inferior vena cava (IVC) dilatation, and moderate mosaic attenuation, without pleuroparenchymal lung disease. Echocardiography was nondiagnostic because of poor sonographic windows. CMR imaging was not performed.

Summary

This five-patient case series highlights a recognizable cardiothoracic imaging pattern among individuals with OHS, comprising enlarged central pulmonary arteries with relative attenuation of the peripheral pulmonary arterial branches, mosaic lung attenuation without pleuroparenchymal lung disease, and variable evidence of right-sided cardiac remodeling or dysfunction. All patients had obesity, OSA, daytime hypoxemia, and daytime hypercapnia, and none had underlying lung disease on CT. These baseline demographic and clinical features are summarized in Table 1. The multimodality imaging findings are summarized in Table 2. These findings suggest possible pulmonary vascular disease associated with chronic obesity-related hypoventilation and warrant further cardiopulmonary evaluation to confirm the presence and severity of pulmonary hypertension.

**Table 1:** Demographic characteristics, clinical variables, and pulmonary function test results of patients with obesity hypoventilation syndrome. *Only two of the five patients underwent echocardiography and/or right heart catheterization.

Variable	n (%)	Mean ± SD	Median (IQR)	Min-Max
Demographics	Missing data: 0/5			
Male sex	3 (60)	NA	NA	NA
Age, years	5 (100)	56.8 ± 19.0	61.0 (39.0, 74.0)	35.0-75.0
BMI, kg/m²	5 (100)	46.5 ± 7.4	44.8 (43.7, 51.9)	36.7-55.5
BMI ≥30 kg/m²	5 (100)	NA	NA	NA
Clinical variables	Missing data: 0/5			
Underlying lung disease	0 (0)	NA	NA	NA
Obstructive sleep apnea confirmed by sleep testing	5 (100)	NA	NA	NA
Daytime hypoxemia while breathing room air, PaO₂ <70 mmHg	5 (100)	NA	NA	NA
Daytime hypercapnia while breathing room air, PaCO₂ ≥45 mmHg (≥6.0 kPa)	5 (100)	NA	NA	NA
Polycythemia	1 (20)	NA	NA	NA
Biventricular heart failure	1 (20)	NA	NA	NA
Pulmonary hypertension diagnosed by echocardiography or right heart catheterization	2 (100)*	NA	NA	NA
Pulmonary function tests	Missing data: 2/5			
Moderate restrictive ventilatory defect without airway obstruction	2 (67)	NA	NA	NA

**Table 2:** Imaging findings in patients with obesity hypoventilation syndrome. RV: Right ventricle; LV: Left ventricle; IVC: Inferior vena cava; LVEF: Left ventricular ejection fraction. The CT examinations included one contrast-enhanced study and four unenhanced studies.

Variable	n (%)	Mean ± SD	Median [IQR]	Min–Max
Chest Radiograph	Missing data=1/5			
Diameter of the right interlobar pulmonary artery, mm	NA	19.8 ± 2.2	20.0 [18.5, 21.2]	17.0–22.0
Right interlobar pulmonary artery diameter >16 mm	4 (100)	NA	NA	NA
Small peripheral arteries	3 (75)	NA	NA	NA
Dilatation of the central pulmonary arteries	4 (100)	NA	NA	NA
Cardiomegaly	3 (75)	NA	NA	NA
Chest CT	Missing data=0/5			
Lung or pleural disease	0 (0)	NA	NA	NA
Small peripheral arteries relative to central pulmonary arteries	4 (80)	NA	NA	NA
Diameter of the main pulmonary artery trunk, mm	NA	35.8 ± 6.5	32.0 [32.0, 36.0]	32.0–47.0
Main pulmonary artery trunk diameter >29 mm	5 (100)	NA	NA	NA
Pericardial effusion or thickening	0 (0)	NA	NA	NA
Mediastinal lipomatosis	1 (20)	NA	NA	NA
RV:LV size ratio >1	3 (60)	NA	NA	NA
LV dilatation	0 (0)	NA	NA	NA
IVC dilatation	2 (40)	NA	NA	NA
Mosaic attenuation	4 (80)	NA	NA	NA
Transthoracic Echocardiogram	Missing data=2/5			
Diagnostic study	0 (0)	NA	NA	NA
Cardiac MRI	Missing data=4/5			
Diagnostic study	1 (100)	NA	NA	NA
Large main pulmonary artery, normal RV size, decreased RV function, normal LV size, and LVEF 55%	1 (100)	NA	NA	NA

## Discussion

OHS is increasingly recognized as a distinct, underdiagnosed condition with substantial cardiopulmonary consequences [1,2]. Persistent hypoventilation and hypoxemia contribute to pulmonary vasoconstriction, vascular remodeling, pulmonary hypertension, and secondary right heart strain [2,5,6]. These pathophysiological mechanisms may explain the vascular and cardiothoracic imaging abnormalities observed.

Central pulmonary artery enlargement with relative peripheral arterial attenuation was a primary imaging finding. On chest radiographs, this appeared as prominent hilar pulmonary arteries and enlargement of the interlobar pulmonary artery, while CT clearly demonstrated enlarged central arteries with comparatively small peripheral branches. Previous literature identifies central pulmonary artery enlargement as an important imaging indicator associated with elevated pulmonary artery pressures, while radiographic enlargement of the interlobar pulmonary artery supports suspicion of pulmonary vascular disease [7]. The characteristic pruning-like vascular pattern was consistently observed across cases.

Mosaic attenuation was noted in all but one patient and ranged from mild to moderate. Although nonspecific, mosaic attenuation may result from small airways disease, pulmonary vascular disease, or infiltrative lung disease [8,9]. In our cases, the absence of pleuroparenchymal abnormalities and the presence of pulmonary artery enlargement favored a vascular rather than airway or interstitial cause. However, none of the CT examinations included expiratory-phase imaging; therefore, underlying small airways disease could not be excluded. Mosaic attenuation may reflect regional perfusion heterogeneity caused by uneven pulmonary vascular remodeling and hypoxemia in OHS.

Importantly, none of the cases exhibited pleuroparenchymal lung disease. The diagnostic criteria for OHS require the exclusion of alternative causes of hypoventilation, such as parenchymal lung disease [1-4]. The absence of interstitial lung disease or emphysema on CT supports the predominance of pulmonary vascular abnormalities rather than structural lung disease in this series.

Cardiac findings varied but generally indicated right heart strain. Four patients had cardiomegaly on CXR, RV enlargement was observed on CT in three patients, and one patient demonstrated mild RV systolic dysfunction on CMR imaging. Unfortunately, echocardiography played a limited role because of technical limitations related to body habitus. One patient underwent right heart catheterization, which confirmed mild pulmonary hypertension and supported the physiological significance of the cross-sectional imaging findings in that patient. The results of our case series align with previous studies linking OHS to pulmonary hypertension and RV dysfunction [2,5,6].

Pulmonary hypertension was directly confirmed by right heart catheterization in only one patient (Case 4). In the remaining four patients, pulmonary hypertension was inferred indirectly from surrogate imaging markers, central pulmonary artery enlargement, RV enlargement, and mosaic attenuation, rather than confirmed by echocardiography or invasive hemodynamic measurement. Echocardiography was either not performed or was limited or nondiagnostic because of body habitus. These imaging findings should therefore be regarded as suggestive of, rather than diagnostic of, pulmonary hypertension in the absence of confirmatory testing.

Our findings emphasize the complementary roles of multimodality imaging. CXR raised suspicion through a large body habitus, central pulmonary artery enlargement, and cardiomegaly, while CT characterized pulmonary vascular size and morphology, excluded pleuroparenchymal lung disease, and defined RV and IVC size. In one case, CMR provided further information about ventricular function and did not demonstrate alternative myocardial pathology. Integration of vessel-caliber assessment and right heart morphology using multimodality imaging is advisable when pulmonary hypertension is suspected in patients with obesity [6], as supported by our observations in OHS.

Differential diagnoses for enlarged central pulmonary arteries with peripheral pruning and mosaic attenuation include chronic thromboembolic pulmonary hypertension, idiopathic pulmonary arterial hypertension, pulmonary hypertension due to chronic lung disease, and small airways disease [5,7-9]. In our cohort, the combined presence of hypercapnia, obesity, and OSA favored OHS, although it did not exclude these alternative diagnoses (Table 3). Because four of the five CT examinations were unenhanced, chronic thromboembolic disease could not be excluded solely on the basis of the CT findings. PFTs were available for three of the five patients; none showed airway obstruction, two showed a restrictive ventilatory defect, and one had normal results. PFTs were not performed in the remaining two patients (Cases 1 and 5), so small airways disease could not be excluded on pulmonary function grounds in these cases. In these patients, the diagnosis of OHS relied more heavily on the overall clinical constellation of obesity, hypercapnia, hypoxemia, and confirmed OSA, together with the absence of pleuroparenchymal disease on CT. In this case series, the consistent clinical context of obesity, daytime hypercapnia, hypoxemia, and OSA supported the presence of possible OHS-associated pulmonary vascular disease.

**Table 3:** Diagnostic paradigm for enlarged central pulmonary arteries, attenuation of the peripheral pulmonary arteries, and mosaic attenuation on chest CT in patients with obesity. COPD: Chronic obstructive pulmonary disease; V/Q: Ventilation-perfusion; PaO₂: Partial pressure of arterial oxygen; PaCO₂: Partial pressure of arterial carbon dioxide; FEV₁: Forced expiratory volume in one second; FVC: Forced vital capacity; TLC: Total lung capacity; RV: Residual volume; DLCO: Diffusing capacity of the lungs for carbon monoxide; ILD: Interstitial lung disease; ABG: Arterial blood gas; PFTs: Pulmonary function tests; PH: Pulmonary hypertension; FEF_25-75%_: Forced expiratory flow between 25% and 75% of forced vital capacity.

Condition	Expected PaO₂	Expected PaCO₂	Spirometry (FEV₁/FVC)	Lung volumes (TLC/RV)	DLCO	Notes
Obesity hypoventilation syndrome (OHS)	↓ (often <70 mmHg at sea level)	↑ (daytime hypercapnia ≥45 mmHg)	Normal or restrictive pattern	TLC often ↓ (obesity-related restriction); RV usually normal	Normal (extrapulmonary restriction pattern)	ABG definition includes awake hypercapnia with hypoxemia; PFTs may reflect obesity-related restriction.
Idiopathic pulmonary arterial hypertension (IPAH)	↓ (mild-moderate hypoxemia)	↓ (mild-moderate hypocapnia)	Usually normal	Usually normal	↓ (pulmonary vascular disease pattern)	Typical constellation: mild-moderate hypoxemia plus hypocapnia; DLCO is reduced in pulmonary vascular disease.
Chronic thromboembolic pulmonary hypertension (CTEPH)	↓ (often mild-moderate; may worsen with exercise)	↓ (often hypocapnia from hyperventilation/dead space)	Usually normal	Usually normal	↓ (pulmonary embolism/pulmonary vascular disease decreases DLCO)	Precapillary PH physiology; DLCO is reduced in pulmonary embolism/pulmonary vascular disorders.
Asthma	↓ (hypoxemia is common during exacerbation)	↓ early; normal/↑ if severe (fatigue/impending failure)	Obstructive pattern; bronchodilator reversibility is common	RV ↑ (air trapping); TLC normal/↑	Normal or ↑	Hypocapnia is typical unless obstruction is severe, when hypercapnia may occur; DLCO can be increased in asthma.
Small airways disease (e.g., bronchiolitis/early COPD phenotype)	Normal or ↓ (depends on severity; ↓ with V/Q mismatch)	Normal or ↓ early; ↑ in advanced/chronic hypercapnic disease	May be normal early; obstructive pattern (↓ FEV₁/FVC) when established	RV ↑ (air trapping); TLC normal/↑	Often normal (unless emphysema/ILD overlap)	Small airway involvement may show reduced mid-flows (FEF25-75) and air trapping; ABG varies with severity.

Another critical diagnostic dilemma is distinguishing pulmonary hypertension related to heart failure with preserved ejection fraction (HFpEF) from that related to OHS. On chest CT, patients with HFpEF frequently have a mosaic attenuation pattern and enlarged central pulmonary arteries, similar to patients with OHS. However, they may also have left atrial enlargement and smooth interlobular septal thickening. Less common findings include peribronchovascular interstitial thickening and small bilateral pleural effusions [10]. Except for the development of frank right heart failure, these findings are highly unusual in isolated OHS. Hypercapnia and restrictive PFT findings are also distinguishing features of OHS. Finally, patients with OHS do not improve with diuresis.

Limitations of this series include its small sample size and limited availability of confirmatory diagnostic testing, such as right heart catheterization. Only one patient underwent advanced cardiac imaging, limiting our assessment of CMR in this patient population. Four of the five patients underwent only unenhanced CT. Echocardiography was not performed in Cases 2 and 3 and was limited or nondiagnostic in Cases 1, 4, and 5; CMR was performed only in Case 4. Consequently, congenital cardiovascular abnormalities, such as an atrial septal defect or partial anomalous pulmonary venous return, and chronic thromboembolic pulmonary hypertension could not be definitively excluded in all cases. In Case 4, the history of congenital aortic surgery further raises the possibility of an undiagnosed shunt. Notably, no shunt or thromboembolic disease was suspected or reported by the treating pulmonologists in any of these patients; however, in the absence of confirmatory imaging, this cannot be considered definitive. Pulmonary arterial enlargement in this series should therefore not be attributed to OHS with certainty, and these findings should be regarded as hypothesis-generating rather than diagnostic.

The clinical picture in Case 4 was further complicated by a history of congenital aortic surgery, spinal fusion, and pectus excavatum, each of which may independently impair respiratory mechanics or restrict lung volumes. These factors may have contributed to the patient’s restrictive ventilatory pattern and imaging findings independently of, or in addition to, OHS, and their relative contributions cannot be fully distinguished from possible OHS-associated pulmonary vascular changes in this case.

Nevertheless, the central pulmonary artery enlargement and peripheral vascular attenuation observed across all five patients highlight a potentially recognizable cardiothoracic imaging phenotype that may prompt radiologists and clinicians to consider OHS and possible associated pulmonary vascular involvement in the appropriate clinical context. However, associated findings such as mosaic attenuation were not universal, being absent in Case 2, and imaging protocols were not standardized across cases.

## Conclusions

Obesity hypoventilation syndrome may present with a potentially recognizable multimodality cardiothoracic imaging pattern characterized by central pulmonary artery enlargement and relative attenuation of the peripheral pulmonary arterial branches, features present in all five patients, along with variably present mosaic lung attenuation and right-sided cardiac remodeling. Recognition of this pattern in patients with obesity, sleep-disordered breathing, daytime hypercapnia, and hypoxemia should prompt consideration of OHS-related pulmonary vascular disease and further cardiopulmonary evaluation. Additional studies are needed to confirm these imaging findings in a larger patient population, ideally incorporating contrast-enhanced CT to evaluate for shunt and thromboembolic disease, alongside confirmatory right heart catheterization, to establish the true relationship between the degree of obesity and the degree of pulmonary arterial dilation and determine the usefulness of these findings in directing patient care.
